# To Avoid Smoke

**Published:** 1854-12

**Authors:** 


To avoid Smoke.—It is said, that if a silk handkerchief is wetted and placed over the face, a person may pass through dense smoke without inconvenience.

